# Tackling thermosensation with multidimensional phenotyping

**DOI:** 10.1186/1741-7007-10-91

**Published:** 2012-11-19

**Authors:** William R Schafer

**Affiliations:** 1 MRC Laboratory of Molecular Biology, Hills Road, Cambridge CB2 0QH, UK

## Abstract

Most if not all animals sense temperature using specialized thermosensory neurons. Genetic studies in simple organisms have been used to identify gene products required for detecting temperature changes or for mediating the effects of temperature on behaviour. A recent study has used automated imaging and multidimensional phenotyping to characterize behavioural responses to aversive temperature changes and to identify mutants with specific defects in these processes.

See research article: http://www.biomedcentral.com/1741-7007/10/85

## 

Thermosensation, the ability to detect temperature and temperature changes, is nearly ubiquitous among animal nervous systems. Yet the temperature sense remains poorly understood at many levels. In mammals as well as other organisms, specific classes of somatosensory neurons have been identified whose activity is modulated by increases or decreases in temperature. At the molecular level, several ion channels of the TRP family, known as thermoTRPs [1], are gated by thermal stimuli, either by cooling (for example, TRPM8) or warming (for example, TRPV1 and TRPA1). However, it is probable that additional molecular temperature sensors remain to be identified. Moreover, the neuronal circuits and systems that integrate and process thermosensory information are in most cases not well understood.

Studies in simple genetically tractable organisms provide a promising approach to addressing some of these questions. In particular, the nematode *Caenorhabditis elegans *has been used to study thermosensation at the molecular, cellular, and neural circuit levels. *C. elegans *exhibits several distinct behavioral responses to temperature. Perhaps the most intensively studied is thermotaxis, through which animals navigate in a thermal gradient toward their cultivation temperature [2]. Thermotaxis actually involves two distinct behavioral strategies: a biased random walk, in which animals crawling toward the optimal temperature increase their frequency of forward runs and suppress reversals and turns, and isothermal tracking, in which animals at the optimal temperature steer their movement to follow the desired isothem [3]. For both these behaviors, the most important sensory neuron is AFD, a ciliated neuron activated in response to small increases in temperature [4]. Sensory transduction in these neurons requires a cyclic GMP-gated channel; however, the molecules that actually sense temperature in AFD are not known.

*C. elegans *shows a different set of behavioral responses to more abrupt temperature changes from a desirable to a noxious temperature. For example, heating an animal from 22 to 35°C, or alternatively cooling it from 22 to 15°C, evokes a rapid and robust set of escape behaviors, including reversals and large turns [5,6]. Heat avoidance has been shown to require several polymodal nociceptors in the head, such as the FLP and ASH neurons, while cold avoidance depends on a single pair of PVD nociceptors, located in the body. In the PVD neurons, the TRP channel TRPA-1 is required for cold responses, and heterologous expression experiments have identified it as an authentic thermosensor [6]. In contrast, although several TRP channel mutants have abnormal heat avoidance, none has been definitively identified as a thermosensor [5,7]. Clearly many of the key molecules involved in *C. elegans *thermosensation remain to be identified. Moreover, the neural circuit mechanisms by which thermosensory behavior is generated remain incompletely understood, especially with respect to heat and cold avoidance.

Traditionally, the identification of gene products and neurons involved in behaviors such as thermosensation has relied on manual assays with a single readout - for example, a thermotaxis index measuring how well animals navigate toward the cultivation temperature. Such assays can then be used to characterize neural circuits by laser ablation or to screen for behavioral mutants in forward genetic screens. An alternative approach, made possible by advances in machine vision and imaging technology, is to use a high-throughput, high-content assay to characterize behavior in detail and to survey known genes for behavioral phenotypes. A recent study by Ryu and colleagues [8] exemplifies the potential advantages of such an approach.

Focusing on thermal avoidance behaviors, the authors set out to characterize responses to increases in temperature using automated imaging, then used high-content phenotyping to assess the importance of particular genes in these behaviors. As a first step, they used an infrared laser to apply reproducible step-changes in temperature to freely behaving worms, whose movements were recorded by an automated tracking microscope. Analysis of these videos defined a stereotyped sequence of actions - a pause, a reversal, a deep ('omega') turn, and a forward movement - that occurred during the execution of an escape response. Interestingly, although the sequence of motor actions and several motor parameters appeared invariant over a wide range of stimulus strengths, a number of behavioral features, such as the latencies of the state transitions and the speed of the forward acceleration step, varied significantly between animals responding to small and large increases in temperature. The finding that the worms execute different escape behaviors in response to large and small heat steps suggested that *C. elegans *perceives noxious heat stimuli of different magnitudes differently, and that this distinction may rely on distinct molecular thermotransduction pathways and neural circuits.

To investigate this possibility, the authors used their assay to identify strains with abnormal responses to aversive heat stimuli, in particular those with specific sensory (responding only to small or large ΔT) or motor (abnormal in specific steps of escape behavior) phenotypes. The authors surveyed a set of 47 strains with mutations in genes encoding candidate sensory transducers, neuropeptides, or neurotransmission molecules. Each mutant's escape behavior was recorded under four different stimulus conditions (0.4º, 1.0º, 4.8º, and 9.1º) and assayed for 8 features of thermal escape behavior that varied with stimulus strength . Thus, each 15 second recording generated an 8-dimensional feature vector that could be used for comparisons to wild-type and other mutant strains. One comparative approach used hierarchical clustering to identify mutants with similar behavioral patterns in response to particular temperature changes. Strains that reproducibly fell outside the wild-type cluster were inferred to have a significant heat-avoidance phenotype. A second approach used principal components analysis to reduce the dimensionality of the feature data and then identify strains occupying a significantly different region of low-dimensional feature space from the wild-type control. Using this second approach, they identified a number of mutants with significant thermal avoidance phenotypes when tested at small ΔT. Perhaps surprisingly, only a small number of mutants (all with rather general locomotion defects) showed phenotypes measured at large ΔT.

Although this study represents only a first step toward a detailed molecular characterization of aversive thermosensation in *C. elegans*, it has some interesting implications. In particular, since the authors' pilot screen did not identify any genes specifically required for responses to large temperature steps, it is unlikely that known classes of thermosensory molecules can account for all the thermosensory capability of *C. elegans*. The involvement of cyclic nucleotide-mediated signaling in thermotaxis already suggests that thermosensory transduction in neurons depends on sensor molecules other than the known thermoTRPs and potassium channels [4]. The approach pioneered here may provide a powerful means of identifying such novel temperature sensors, which potentially might play conserved roles in the nervous systems of other animals.

More generally, this study illustrates new possibilities for applying bioinformatic techniques to the study of behavior. *C. elegans*, with its simple body plan and largely two-dimensional repertoire of movement, is ideally suited to the use of automated imaging for quantitative phenotyping, especially with regard to behavior. Previous studies have demonstrated that parameters extracted from *C. elegans *video data can identify functional links between nervous system genes based on clustering and projection into multidimensional feature space [9,10]. However, this earlier work focused on the behavior of mutants with grossly uncoordinated locomotion in constant environmental conditions, and therefore did not effectively assess aspects of nervous system function related to sensation. This study demonstrates that even short recordings of stimulus-evoked behavior can generate high-content data for assessing sensory abnormalities at relatively high throughput. It is not unreasonable to envision using such methods on a much larger scale; indeed, a whole-genome phenotyping effort for *C. elegans *seems a realistic prospect.
